# Book Reviews

**Published:** 1829-05

**Authors:** 


### CRITICAL ANALYSES.

Quæ laudanda forent, et quæ culpanda, vicissim

Illa, prius, cretâ; mox hæc, carbone, *notamus*.—Persius.

*A Treatise on Obstructed and Inflamed Hernia; and on Mechanical Obstructions of the Bowels internally; and also an Appendix, containing a brief Statement of the Cause of Difference in Size in the Male and Female Bladder.* By Henry Stephens, Member of the Royal College of Surgeons.—8vo. pp. 191. E. Cox, London, 1829.

Mr. Stephens informs us that the present volume originated in the following manner: A case accidentally fell under his care, which exhibited certain symptoms and features not altogether corresponding with those ideas and that information which he had derived from the best authorities. Hence arose in his mind a train of reflections and inquiries, which have proved highly instructive to himself, and which he trusts may be useful to others if more publicly known. The paucity of cases in illustration of his doctrines which the author has given in his work, arises not from want of corroborative materials, but from an unwillingness to increase the size of his book.

It has not been the purpose of Mr. Stephens to write a complete treatise upon the subject of hernia generally, but only to supply what his investigations have induced him to believe were important omissions or defects in the works of previous authors, and to describe some varieties of the disease which have not yet been noticed. This work may, therefore, be considered as an appendage to existing treatises upon hernia.

The first division of the volume treats of "*Obstructed Hernia.*" Long-existing and irreducible hernia, says the author, produce many remarkable and painful symptoms, and even death, from a cause which it is in the power of surgery to remove by an operation, when the true nature of the malady is well understood. A remarkable case of this kind came under his care, in which an operation was successfully performed, "although it was one of those cases which have not been considered as requiring it, no strangulation existing."

The patient, a woman, was attacked with sickness and pain in the bowels. She was at first suspected to be threatened with cholera, which was then prevalent. For several days she had no motion, although aperients and injections were freely employed. After this time the matter vomited had a fecal appearance, with a very offensive smell. There was no tension, and but a slight soreness of the abdomen. The croton oil was given: this, however, like all the other aperients, only increased the vomiting. She said that she had no rupture. On the fourth day her countenance began to exhibit signs of sinking, and the pulse was getting feeble and fluttering. These symptoms slowly, but progressively, increased. A week after the author first saw her, she slightly mentioned a swelling on the side of the abdomen, which had existed twenty years. It was found to be a ventral hernia, having upon its surface an old cicatrix. It was evidently not strangulated. It was not tense nor painful upon pressure. It receded under the touch, and passed readily into the abdomen with a gurgling noise, but returned when the pressure was removed. The pain was not of the character which indicates strangulation of a portion of intestine. The symptoms were not, therefore, referred to the hernia. She continued sinking, had occasional faintings, hiccup, and still stercoraceous vomiting. She had been long subject to a complaint in her bowels, which had been thought to be colic. Reflecting upon the history of the case, Mr. S. concluded that the symptoms, although not those of strangulated hernia, were yet such as would be produced by any permanent and mechanical obstruction in the bowels, and that it was highly probable that the obstruction was in that portion of the bowel which was contained in the hernial tumor. He therefore determined to cut into the hernial swelling, and examine the condition of the parts, and thus see if relief was possible. With some difficulty the patient consented to the operation. Mr. S. divided the integuments, which were very thin, by a crucial incision, and afterwards a superficial fascia, with some cellular structure. The hernial sac was now exposed and opened freely. A portion of small intestine was found within, which was irreducible; another portion being loose, and readily passing into the abdomen when pressed upon. The small irreducible knuckle of intestine was adhering very firmly to the hernial sac, and in a position which at once accounted for the previous symptoms. It was so closely united by adhesions to the hernial sac as to obstruct, to all appearance, its peristaltic action, and prevent the due course of its contents. There was no stricture; for the operator easily passed his finger into the abdomen by the side of the intestine, which was somewhat discoloured. The bowel was relieved from its adhesions to the sac, and pushed into the abdomen; the finger being passed in and around the opening on the inside, to determine that there was no further adhesion. The wound was then sewed up. The patient did not feel that immediate relief from the operation which is experienced in cases of strangulated hernia. The first favorable symptom that occurred was her swallowing some liquid, without vomiting being produced, which had not been the case before the operation. In two hours she passed some fecal matter from the bowels, for the first time since her illness, and the hiccup had much abated. Her countenance was less anxious; pulse still low and fluttering; less sensation of sinking. She continued for three days slowly improving. On the third day she had copious motions, from aperient medicines: the pulse after this immediately began to rise, the sense of sinking almost instantly went off, and she rapidly recovered.

"The necessity, or at least the utility, as Sir Astley Cooper has always indicated, of freely purging a patient after an operation for hernia, is in this case remarkably shown: the sense of sinking, and the alarming depression of the vital powers, are the effect of a suspension of the natural function, and, until there is a resumption of the peristaltic action, the patient does not thoroughly revive. The administration of a brisk purgative, and a copious evacuation from the bowels, appeared almost instantly to remove the sense of sinking and depression of the pulse: yet I should think the restoration of the function of the bowels is always quicker after an operation for strangulation, than after an operation for what I call 'obstructed hernia,' because in the former the previous suspension will have been of shorter duration." (P. 8.)

The slow progress and less degree of violence of the symptoms in the case related by Mr. Stephens, prove that it was not one of strangulation. He believes that fatal consequences from adhesion of the bowel to the hernial sac are by no means uncommon, particularly in umbilical and ventral hernia. To confirm the propriety of giving purgatives after the operation, the author adduces the opinions of Sir Astley Cooper. It is of the utmost importance that *such* authority should not be imperfectly quoted. It is true that Sir Astley does recommend purgation after the operation, but he couples this advice with a very important caution, which Mr. Stephens should not have omitted. In speaking of the treatment after the operation, Sir Astley says,* "You order the patient to keep the horizontal pos-

* Sir Astley Cooper's Lectures on Hernia.

ture, but, above all, direct that the evacuations are passed on foul linen, and that he be not allowed to get up. If strict orders be not given to this effect, the patient will get up to go to stool, and great mischief will most probably be done by the exertion. Mr. Cline operated upon a patient in this hospital (St. Thomas's) for strangulated hernia, and the parts were returned to the abdomen; but the patient got out of bed a short time after the operation, and, when on the close stool, the parts descended into the sac, and displaced the dressings. Mr. Cline was sent for, who found the hernia as large as before the operation. He reduced it, and ordered that the patient should not quit his bed. I mention this case to point out to you the necessity of enjoining the horizontal posture."

Mr. Stephens mentions another case, still further to strengthen the opinions he derived from that of which we have given an abstract. The symptoms in this instance were somewhat obscure. There was pain in the abdomen, vomiting, and obstinate constipation. Purgatives and injections were employed without avail. Still no danger was indicated.

"I conjectured this to be a similar case to the one before mentioned, and immediately inquired if she had been previously subject to pain of the bowels, or colic, after taking her meals? To this she replied in the affirmative, which was also attested by her attendants, who informed me that she had often been obliged to leave the table from pain in the bowels. She had never, to her knowledge, been the subject of rupture. I then proceeded to examine the abdomen, the integuments and parietes of which were loaded with fat. I examined carefully every part, and found various prominences from accumulations of fat; but at one part, a little below the umbilicus, one of these prominences appeared larger than the rest, and somewhat different to the touch, but conveyed no certain indication. The professional gentleman in attendance soon after came, and gave me privately a history of the case from the time of his being called in. I inquired, without informing him what I thought, his opinion of its nature. He considered it a kind of inflammation of the bowels, but at the same time said it did not present the symptoms of genuine enteritis. He had considered the case as one of somewhat unusual character. I then gave my opinion that it was a case where, if we could discover the proper part, an operation would relieve; and I told him the case I had formerly had, and my reasons for supposing this to be similar." (P. 14.)

It was agreed that an operation would not be justifiable without further evidence of the precise nature of the case. It was doubtful even whether hernia existed, and as yet there was no immediate danger. Four days after Mr. S. first visited the patient, the matter vomited was feculent. Every thing was thrown from the stomach, and no evacuation could be procured from the bowels, and the use of aperients was given up, as they only increased the vomiting. Injections were tried, and endeavours made to calm the irritability of the stomach by anodynes. Although the patient was evidently sinking, the symptoms were not immediately alarming. An operation was proposed. The patient and her friends were anxious to delay it, and it was determined to operate the succeeding day, if no amendment took place. She was then, however, in a state which precluded all hope of saving her. The author thus describes the dissection of the case.

"I explained to my colleague that I expected to find the bowels confined by adhesions, in some position unfavorable to the passage of its contents. I accordingly cut down upon the part where I supposed the hernia, if any existed, would be found; and, after dissecting, with the assistance of my colleague, to a very considerable depth, through cellular structure and fat, discovered the sac of a hernia: upon cutting through this, a circumscribed cavity was seen, containing at the bottom a small portion of intestine. Upon passing my fingers into this cavity, I found a canal leading from it obliquely towards the umbilicus; I passed my finger along this sinous canal, by the side of the intestine, and at length through the umbilicus, into the abdomen. Here, then, was a hernia, which had caused death without any stricture or strangulation. The portion of bowel which had escaped the umbilicus, and insinuated itself obliquely under the fat and integuments, was closely adhering to the sac, and doubled upon itself, so as effectually to obstruct its peristaltic action and the passage of its contents. After we had satisfied ourselves that no stricture existed, and that the cause of death was simply a mechanical obstruction, we separated the bowel from the strong adhesions by which it was held, and passed it readily into the abdomen. We then opened the abdomen, and examined the intestines, and found that portion which we had just returned of a dark colour, but not in the least gangrenous. The intestinal canal leading from this portion was discoloured for three or four inches, but much less so than the part itself. The bowels generally exhibited no marks of inflammation, nor did the peritoneum. My colleague was now, as well as myself, perfectly satisfied that if the patient and her friends had consented to an operation the day previously, her life might have been saved; and also that death from adhesions of the bowels in hernia may take place, as surely as from strangulation." (P. 18.)

In reading the account of the dissection in fatal cases of hernia after operation, the author has often noticed, amongst other morbid appearances which have been detailed, that there were found adhesions of the bowels, and that a knuckle of intestine is often described as so adhering.

"In operating for strangulated hernia, therefore, the surgeon should not consider that he has done all that is required when he has divided the stricture: he ought not to return the bowels until he is satisfied that they are so freed from their adhesions that, when returned into the abdomen, they will be capable of resuming their functions. Upon this principle I venture to differ from Sir Astley Cooper, who directs, in the operation for large hernia, that the stricture should be divided without opening the sac. If the symptoms are such as to leave no doubt that they are caused by a stricture solely, then the above proceeding is certainly preferable; but as I believe that in many of these cases dangerous adhesions exist, I cannot avoid recommending that the sac should, in all doubtful cases, be opened, the state of the parts within examined; their adhesions, where it is practicable, removed; and the contents of the hernia, if possible, returned into the abdomen." (P. 20.)

It cannot, we must observe, be doubted that Sir Astley Cooper would open the sac "in all doubtful cases;" for he particularly remarks, in his Treatise on Hernia, that he has known a fatal obstruction to the passage of the intestinal matter to arise from the mere adhesion of the two sides of a fold of intestine together. It is now, indeed, well understood that every preternatural connexion should always be separated before the viscera are reduced.* In the case of John Harris,† which is related in our Journal, in which Mr. Earle operated, it is expressly stated that the protruded intestines were inseparably connected with the surrounding parts. In commenting upon this case, Mr. Stephens remarks, that he does not consider that any blame can reasonably attach to Mr. Earle, "the existing knowledge upon the subject not having distinctly shown that symptoms resembling strangulation can be caused by adhesions only, and that relief can be given by a simple separation of the adhering surface." The fact is, that neither Mr. Earle, nor the "existing knowledge" at the time, can be complained of. If Mr. Earle *could* have separated the adhesions of the intestines from the surrounding parts with safety, he would of course have done so, in compliance with the admitted principle, to which we have before referred, of separating *every* preternatural connexion, if possible. We recommend Mr. Stephens to read once more Mr. Earle's detail of Harris's case. He will find that Mr. E. came to

* Lawrence on Hernia, p. 153, first edition.

† London Med. and Phys. Journal, Nov. 1827, p. 417.

the same conclusion that he does, namely, that it was a case where the adhesions and morbid connexions of the parts produced a total obstruction of the natural function and action of the bowels."

As the author has introduced this case for the express purpose of entering into a critical examination of it, he ought not to have abbreviated it. The impression that would be created as to Mr. Earle's opinions of Harris's case, from the account which he has himself given, must be very different from that which would arise from the perusal of the imperfect abstract given of it in the volume before us. The author states "that the division of a stricture is supposed to be all that is positively required in the operation for hernia, may be collected from all authors who have written upon the subject." And again he observes, "*I* believe that two causes of danger may exist at the same time, namely, stricture and adhesions, and that, unless both are attended to, the safety of the patient is not ensured." Now, the division of a stricture is *not* supposed to be all that is required; for, as we have already stated, the surgeon has long been taught to make it a rule to destroy every preternatural connexion before he returns the part, because the "two causes of danger," namely, stricture and adhesion, were well known, and their importance duly appreciated.

"*On Inflamed Hernia.*"—The contents of a rupture are said sometimes to become inflamed, in connexion with an inflammation of the bowels generally, and totally independent of any cause arising from the rupture. Mr. Stephens believes such instances are rare.

"These inflammations, I believe, are almost always generated by the morbid condition of the parts within the rupture, and afterwards becomes quickly communicated to the interior of the abdomen. Large irreducible herniæ, more especially umbilical, are those in which this form of disease mostly occurs, which appears to partake more of the character of enteritis than of ileus. A small portion of confined intestine, however intensely inflamed in itself, does not so necessarily or so quickly communicate its disease throughout the abdomen, it being of comparatively local origin; but, when the contents of a large hernia become inflamed, as a sequel (I believe) of various chronic confinements and changes of structure in the parts, the disease from the first will be of a more diffused and general character, and will more extensively and quickly communicate with the interior.

"Although large irreducible ruptures are those in which disease and inflammation, independent of mere mechanical obstruction, are most likely to arise, yet small irreducible ulcers are also very subject to this form of complaint, particularly omental, or those wherein omentum is contained. The omentum is subject, in its unnatural situation, to become thickened and diseased, and to suppurate. The hernial sac will also often inflame and suppurate. The appendiculæ epiploicæ of the colon will also occasionally undergo some alteration of structure, when confined within a hernial sac. All these various changes and states of disease become a frequent source of inflammation to the contiguous bowels or peritoneum. The inflammation which is thus produced is attended by an obstinate obstruction and symptoms of general inflammation throughout the abdomen, and is generally fatal in its consequences. When the herniæ are small, the inflammation of the rupture, denoted by pain, soreness, and tension of the part, so clearly precedes the inflammation of the abdomen, that the case is usually mistaken for strangulated hernia; and, if an operation is performed, the tension which the parts have acquired fills up the opening through which they have descended, and favors the mistaken opinion of the existence of a stricture. When the herniæ are large, the inflammation of the abdomen and of the hernia are very often nearly simultaneous, and if, upon operating, there is found a palpable absence of stricture, then the hernia is supposed to be merely participating in a general inflammation of the intestines. The want of success attending operations upon large herniæ, particularly umbilical, is attributed to the direct exposure of the peritoneal cavity, by which a dangerous inflammation is excited. I believe that the inflammation which destroys the patient is, in the majority of cases, altogether established before any operation is attempted." (P. 68.)

Cases of unsuccessful operation for hernia are, in the author's opinion, very frequently of the above kind.

At pages 36 and 37, part first, of Sir Astley Cooper's work, a case is related, which appears to Mr. Stephens to be of that kind which he terms inflamed hernia.

"'A woman was admitted (into Guy's Hospital, 1803,) with three herniæ, two in the groin and one at the navel. The umbilical hernia and that of the left groin were irreducible; that of the right groin felt extremely sore upon pressure. A doubt arose which was the hernia that required the operation; but, as the symptoms of strangulation were not extremely urgent, though the woman was very low, it was agreed to wait till the next day for a consultation. During the night, however, she died, and, upon inspecting the body, the tumor in the right groin was found to be an enlarged and inflamed absorbent gland, lying over an empty hernial sac. In the left groin was a portion of inflamed intestine; and at the navel was an irreducible omental hernia, which had suppurated, and contained about a tablespoonful of matter.'

"This woman complained chiefly of pain in the right groin, and if the operation had been performed, this would have been the tumor laid open. This case also furnished another observation:

though this woman had several herniæ, yet the operation, on whichever it had been performed, would have given no relief, as she died, not of strangulated hernia, but of peritoneal and omental inflammation. When the abdomen was opened, the intestines were found adhering to each other, with matter interposed in some places; and a considerable quantity of pus had been effused into that part of the omentum which was contained in the cavity of the abdomen. In this case, therefore, the abdomen was first affected, and the inflammation, after having extended through it, was continued to the protruded parts. Soreness of the abdomen, therefore, which in strangulated hernia is a late symptom, must here have been one of the earliest." (P. 72.)

The author thinks there can be no doubt that the above was not a case of strangulated hernia, but one of peritoneal and omental inflammation; but he does not agree with Sir Astley in supposing that the abdomen was first affected: on the contrary, he believes that the herniæ generated the inflammation, which was communicated quickly, or otherwise, according to circumstances, to the interior of the abdomen.

"In inflamed hernia, the viscera of the abdomen are very extensively inflamed throughout. In obstructed hernia, very slight traces of inflammation are in general visible after death. Cases of strangulation are of an intermediate kind; the inflammation being almost wholly confined to the seat of stricture and the parts above it, the intestines below being in a state of collapse and uninflamed.

"An empty hernial sac is not unfrequently, by becoming thickened and diseased, a source of inflammation to the bowels and peritoneum; but I have reason to believe that the inflammation so produced is not generally so extensive or so fatal as when intestine is contained within. Coagulable lymph or pus forms within the sac. If the former, adhesive inflammation only has prevailed, and the patient will not unfrequently recover. When pus has formed, the case is more dangerous. An operation appears to do good, by giving exit to any pus or fluid which has been secreted." (P. 89.)

*Treatment of hernia.*—Mr. Stephens believes that, in general, before the actual protrusion of a rupture, there are some sensations which indicate a disposition to it.

"The patient feels at that part, during the action of the abdominal muscles, more especially in evacuating either the bladder or the bowels, a sort of bulge or pressure of the intestines, more on one side than the other, and which bulge becomes gradually a more distinct sensation. In this state of the disease I believe that its further progress may often be checked. I should recommend the person so affected to wear a belt round the lower part of the abdomen and loins, which belt should be supported by straps over the shoulders, in such a manner that the abdomen may be supported or lifted up: at the same time, care should be taken that no part of the dress is worn tight round the upper part, so as to press the contents against the situation of the threatening hernia.

"Where hernia has actually occurred, the patient should in due time make use of a truss, and, by attention to its proper adjustment and constant application, prevent, if possible, the hernia ever protruding. It should be impressed upon the patient's mind that a rupture produces no injury if it is kept from descending, but that, if it is suffered to remain down, it may become strangulated, or may contract adhesions and become irreducible." (P. 91.)

We fear the preliminary symptoms of hernia are not sufficiently indicative of the nature and importance of the threatened evil to induce the patient to apply for surgical advice, without which the proper precautionary mode of treatment will not be adopted.

When a rupture has become irreducible, the patient must endeavour, if possible, to prevent its further increase. Every contrivance in the shape of a truss, to make pressure on the ring, must be particularly avoided. An elastic bag truss will form the most proper support. "It should be so contrived as to close upon and grasp the lower part of the tumor, by which means it will lessen its bulk by promoting absorption, and render the protrusion of fresh parts more difficult. Care must be taken that the bandage makes no circular pressure round the neck of the tumor, as to contract this part would endanger the occurrence of strangulation, and give a disposition to unfavorable adhesions and ultimate obstruction." The patient should also, by exercise and moderate diet, prevent accumulations of fat. In the early stages it is probable that, by confinement in bed and spare diet, with pressure on the base of the swelling by an elastic bag truss, an apparently irreducible hernia may be reduced.

"Instances have occurred, and are related by most writers, where, from confinement to a bed from other causes, herniæ, which have been previously irreducible, have of themselves gone up; particularly scrotal herniæ.

"When a person has an irreducible rupture, which has given indications of a tendency to obstruction, by producing pain after eating, &c. great attention to diet is required. The food should be well masticated, and so reduced and divided as not to be likely to obstruct the passage from its solidity or size. The food taken should be as much as possible of a fluid nature, as animal broths, soups, &c.; also light puddings. New bread, indigestible fruits, &c. should be avoided. When obstruction of the bowels has occurred, it is not at once to be considered as irremediable, because many temporary impediments to the passage of the feces occur before a total obstruction is established. Sir Astley Cooper says, 'umbilical hernia often has symptoms of strangulation, subsiding and returning.' I should rather say, symptoms of obstruction. These states of disorder may very frequently be removed by mild laxatives, as the saline purging salts, &c. Calomel and opium, by quieting the disturbance of the intestines, will facilitate the passage of the contents. Bleeding and warm bathing would without doubt be serviceable in allaying the irritation. The successive returns of these symptoms are generally of increased severity, and at length a total obstruction takes place, which resists all the usual means, and leaves the surgeon no other resource than an operation to remove the adhesions and return the bowels." (P. 93.)

In old large herniæ, which are irreducible from want of space in the abdomen, and which are the subject of inflammation, the author does not promise benefit from operating, but he says,

"If a case were to arise in my practice of the contents of a hernia, irreducible from a state of adhesion only, becoming inflamed, believing as I do that such inflammation is produced by, or depends upon, certain morbid conditions and connexions of the parts in the rupture, I should not think I fulfilled my duty to my patient unless I proposed (upon the failure of other means) an operation to render the hernia reducible.

"Most surgeons will probably prefer to place their dependence upon bleeding and measures of general depletion, but I cannot conceive that such means ought to be relied on, while the cause which generated the inflammation still remains unrelieved. When the preternatural condition and connexion of the parts within a rupture is in some degree removed, then depletion may produce some salutary effect. In the opinion of most surgeons, cutting into parts already in a state of inflammation would be the most likely way to increase it; but I do not think that this is by any means a necessary consequence. Where there is determination of blood to a part, incisions, as is the case in erysipelas, have a tendency to relieve. But, admitting that operations upon a part in a state of inflammation have a tendency to aggravate such disease, yet I think it brings the parts into such a state as will allow of relief: whereas the other alternative resigns them to struggle against impossibilities. Who would refuse to operate upon a strangulated hernia, from an apprehension of increasing the existing inflammation? Do we not know that, by removing the cause, we take the most effectual way of subduing the effect? And, although I believe the chance of success is much more remote in cases of simple inflamed hernia, than in cases of obstruction or of strangulation, yet I think that cases may be selected where an operation would be effectual.

"The cases where it might be successful would be those in which the herniæ were not of extreme size, and admitted of reduction easily after the adhesions were separated. The earlier, too, the operation was resorted to, the greater would be the chance of success." (P. 97.)

The author does not conceive there would be much difficulty in distinguishing inflammation of the bowels, which commenced internally, from that which commenced in a hernia: "the pain, swelling, and tension would be in the belly, and not in the rupture, or at least not until a late period; while, in inflamed hernia, these effects would be primarily and principally there."

Mr. Stephens' proposal "to make an immense artificial hernia," to prevent inflammations and obstructions in large irreducible umbilical herniæ, is not likely to be adopted.

In the next section some remarks are offered respecting an operation for returning an irreducible hernia, and "a probable method of radical cure" is proposed. Mr. Stephens says, that "operations upon hernia are not considered necessary or justifiable by surgeons of the present day, unless strangulation has occurred." This statement is not correct. Strangulation is considered the most common, but *not* the only, cause for operating. The following extract from an excellent article upon hernia, by Richerand,* is sufficient to show that Mr. S. is mistaken upon this point. Abundant evidence might be brought from English and foreign writers to prove that the practical point to which the author alludes has not been, as he imagines, overlooked or entirely neglected. Richerand, in speaking of the symptoms arising from intestinal obstruction from the accumulation of fecal matter in the protruded gut, remarks, "Au moment où les symptômes de l'inflammation se joignent à ceux de l'engouement, la tumeur devenant tendue, rénitente et douleureuse, *l'opération se trouve positivement indiquée, et tout retard devient funeste.* Avant de dire comment on y procède, parlons de l'étranglement; c'est la cause qui, *presque toujours,* rend cette opération nécessaire."† Richerand thus clearly defines the distinction between obstruction and strangulation:§ "Dans l'engouement *il n'y a point* étranglement, ou constriction exercée par l'anneau sur la portion d'intestin à laquelle il donne passage: il n'existe pas de disproportion entre cette ouverture et les parties qu'elle embrasse. Bien plus, l'engouement s'établit l'anneau restant dans un état remar-

* Dict. des Sc. Med. tome 21, art. HERNIE.

† Loc. cit. p. 142. § P. 144.

quable de dilatation et de relâchement, sur des viellards affligés de hernies anciennes et volumineuses."

We believe that, amongst the general body of the surgical world, many of whom are imperfectly acquainted with operative surgery, there may be an opinion that strangulation *is* the only cause which can render an operation necessary; but every well-informed practical surgeon is quite as well aware as Mr. Stephens that occasional exceptions are sometimes met with. He, perhaps, would operate earlier in cases of "engouement," or "obstruction," than other surgeons; and upon this point his remarks appear very judicious.

"If a patient with irreducible rupture should be troubled with frequent and considerable intestinal derangements, as vomitings, colic, and pain after taking meals, with frequent obstructions of the bowels, which symptoms appeared to be gradually increasing in violence, I should consider such a state, although not indicating any immediate danger, yet as infallibly denoting such a state of adhesion and change of the parts contained within the rupture as to believe that the time was not far distant when an operation would be absolutely necessary to save the patient from falling a victim to a mechanical obstruction. Under these circumstances I should think it right to propose an operation, which would have the effect of permanently relieving those painful symptoms, and of removing from him an impending danger." (P. 106.)

In performing an operation for the relief of an irreducible adherent rupture, many circumstances require to be duly considered,

"and a degree of selection with regard to the particular case is necessary; for, if an indiscriminate recourse to an operation in irreducible hernia were ever to become a general practice, its want of success would soon bring it into discredit and disuse. The peculiar success which has attended some surgeons in their operations, has not arisen so much from superior skill in operating, as from a more judicious selection and consideration of the particular case requiring it. If the hernial swelling is of great bulk, and it is probable that, when the adhesions are separated, the hernia could not be returned, from the want of capacity in the abdomen to receive it, or from the morbid alterations which it has undergone, the operation should not, except from a certain and immediate apprehension of the loss of the patient's life, be attempted. But if the hernia be small, and be in part reducible, and yet give rise to symptoms of increasing derangement and obstruction, then the operation will be justifiable, and will afford the fairest prospect of success." (P. 109.)

To effect a radical cure, Mr. S. proposes an operation, differing in a very essential point of view from any hitherto proposed, and which he believes affords more probable grounds of answering the intended purpose. He has performed it upon the brute subject with complete success, and he infers it would be equally successful in the human.—The following is an abridged account of the proposed operation:

A friend of the author's had a pointer bitch, the subject of an enormous hernia, which, from its size and weight, rendered the animal nearly useless. Mr. S. began by reducing the condition of the animal, as he foresaw that the less superfluous fat there was upon the omentum and in the interior of the abdomen, the easier would the parts be returned and retained. The operation was begun by feeling for the opening through which the intestines protruded. It was found in the situation of the inguinal ring. An incision was begun directly over it, carrying it about half-way down the surface of the tumor, and through the integuments. A quantity of fine cellular structure was then cut through, and the hernial sac opened: omentum and intestines were found within. The parts were drawn up from the bottom of the tumor, and pushed with the finger through the opening into the abdomen. One considerable portion did not admit of reduction, owing to its strong adhesions below. It was the omentum and one portion of intestine only which were returnable; another portion, being firmly connected to the parts out of the abdomen, had never admitted of reduction. Mr. Stephens proceeded by inverting the hernial tumor, by which means he could see the whole irreducible part of the intestine, without the necessity of laying the sac open to the bottom. The bowel was not simply adhering to the hernial sac; its coats were absolutely incorporated with it, having no line of separation. To attempt, in this case, to dissect the bowel away from the sac would have been hazardous; but it occurred to the operator that he could separate the sac from the integuments, &c. forming the hernial pouch, to which it had become closely joined. He succeeded, and returned the intestine and sac into the abdomen, adhering as he found them. The opening from the abdomen was so considerable, that, unless the finger was constantly there, the parts protruded. To retain the bowel in the abdomen was now the difficulty. A bandage was of no use, and the object was to obtain a radical cure by effectually closing the abdominal opening. The parts were prevented from protruding by the quilled suture, substituting for quills pieces of wood. The immediate return of the hernia was thus prevented; and the remaining part of the wound was closed by sutures. The pressure of the quilled suture upon the vessels of the thigh obstructed the passage of the returning blood, and caused œdematous swelling to some extent in one limb. Incisions relieved this symptom. At the end of four or five days, the sticks and ligatures were removed. The animal now rapidly recovered. The operation was performed in August, and the bitch was used during the shooting season of September, and proved equal to any exertion.

The radical cure in this case appeared to be owing to the return of the hernial sac. It was separated from a very close adhesion to the hernial pouch, and it was returned in this state of recent separation into the abdomen, ready to attach and unite itself to any surface to which it was opposed. By its inclination to descend again, it was, although kept in the abdomen, closely applied to the abdominal ring, over the interior of which it without doubt closely adhered, and thus completed, in an unexpected manner, the radical cure.

From the success attending this really interesting case, the author recommends that, in all cases, the sac should, if possible, be returned, to give a better chance of radical cure. By leaving the hernial sac, a direct channel of communication is preserved between the abdominal cavity and the situation of the hernia. By returning it, besides the probable chance of its adhering over the ring, and thus effectually closing it, we produce a somewhat broken communication between the exterior and interior parts. Care should be taken to keep the patient in such a position that the returned bowels, with the sac, may be applied over the interior of the abdominal ring, at the same time that a fresh descent of the parts is sufficiently guarded against.

In the other sections, Mr. Stephens treats of mechanical obstructions of the bowels within the abdomen, and of the symptoms denoting such obstructions, and the probable signs distinguishing their different varieties and situations. The treatment in the early stages of mechanical obstruction of the bowels is also considered.

A few brief remarks on the cause of the difference in size of the male and female bladder, terminate the volume.

Mr. Stephens may fairly claim the merit of having achieved the object he had in view, of giving a useful appendix to the more general and elaborate treatises on Hernia. There is certainly, however, much less of novelty in many of his doctrines than he himself appears to imagine.

gine. It is proper, however, to add, that many practical points are by him definitely determined and ingeniously commented upon, which, although not neglected by previous writers, had been but imperfectly considered.

---

*A Preliminary Dissertation, illustrative of a new System of Pulmonary Pathology, supported by a Series of conclusive Physiological Experiments; combining a rational Theory with a successful Method of conducting the Cure of Consumption.* By P. P. P. Myddelton, M.D. &c. Author of a Treatise on the Diagnosis and Prognosis of Diseases, an Essay on Gout, and Clinical Reports of Select Medical Cases, with Practical Notes. —8vo. pp. 95. Bath. Baldwin, Cradock, and Joy, London, 1825.

It appears that this preliminary dissertation was delivered by the author, as a lecture, some years ago, in America, and that the practitioners of that country thought very highly of its doctrines. It gives us a brief outline of the author's theory, and a description of the practice which is founded upon it, as well as a concise view of his arrangement of pulmonary consumption under five distinct heads, with some reference to collateral considerations of great practical importance. The arrangement proposed by Dr. M. of pulmonary consumption is as follows:

"1st. The hereditary or constitutional disease, arising from causes of remote origin.

"2dly. The acquired disease, as communicated to persons in the full possession of health, who are not predisposed to pulmonary derangements.

"3dly. The primary disease in combination with other visceral affections.

"4thly. That modification in which the digestive organs are the *primary*, and the pulmonary organs the *secondary*, or sympathetic source of morbid action, which may be truly termed *dyspeptic phthisis.*

"And, lastly, That form of the disease which may be produced by *casual* causes." (P. 4.)

In this classification is included those catarrhal affections arising from inflammation of the larynx and mucous membrane of the bronchial tubes, which often assume all the external characters of confirmed phthisis: "and so indeed with chronic inflammation of the pleura; for that membrane undergoes a progressive change of structure, from a simple effusion of lymph, until, by continued depositions and thickenings, it acquires the vital property of exhalation, and often terminates in empyema."

This passage includes an error both of theory and fact. The pleura does not "acquire" by disease the "vital property of exhalation." In common with all other membranes, a vapour or fluid is constantly exhaling from its surface in a healthy state. From inflammation of the pleura, pus may be thrown out, and the disease termed empyema be formed; but in this morbid process no vital property is "acquired:" the natural functions of the part are merely changed.

"Hereditary or constitutional consumption is, by much, the most prevalent form of the disease, and the product of a strumous diathesis, generated, for the most part, by past generations, and by various causes. It is, nevertheless, a fact of practical notoriety that we occasionally meet with patients, at different periods of life, affected with scrofulous tumors on the external surface; while we meet with others who exhibit strongly-marked symptoms of phthisis pulmonalis, without being able to trace the origin of either disease to any relative source. I must, however, remark, that even a change of location, from an elevated country and pure air to the exhalations of a low marshy situation and humid atmosphere, by deranging the gastric secretion, and impairing the energy of the digestive functions, will induce defective absorption of nutritious particles of food from those organs; a consequent flaccidity of the muscular fibre; and predispose persons so situated to glandular obstructions, which an impoverished diet, impure water, and mental anxiety, would still further increase. In corroboration of this opinion, I can state, from my own personal observation, the fact that several English and Scotch families of my acquaintance, who had removed to Holland with commercial views before the revolution, in a few years became martyrs to scrofula, from the operation of causes already recited; for they had previously exhibited no symptom of glandular or cutaneous disease: and it is worthy of being recorded, that the junior branches of those families were the least sufferers by that disease until they had approximated to the age of puberty; they then became affected with glandular tumors, or chronic pulmonary disease, and in some instances both existed at the same time, and were simultaneous in their progress: the majority of cases, however, it must be admitted, were purely scrofulous. I am, nevertheless, quite aware that the converse of those appearances is more usual in the real hereditary scrofula, where we often see the disease exhibit itself in early infancy. From a residence of several years in Ireland, with ample opportunities for observation, I have remarked that her population is more subjected to scrofula and pulmonary consumption, even in the more elevated ranks of life, than any other section of the united kingdom with which I have had intercourse; and I am led to infer that we may trace the cause of the calamity to the extreme humidity of the atmosphere, occasioned by the exhalations from the numerous large lakes and extensive bogs with which that hospitable and fruitful country abounds; and, among the labouring class of the community, we may, unfortunately, add an impoverished diet, and an habitual disregard of cleanliness, by which the exhalents of the skin are interrupted in the free performance of their appropriate functions, and the subcutaneous glands subjected to congestion or morbid sympathies, by the partial retention of the noxious excreta. So, in northern latitudes, the intensity of cold, without judicious covering, will induce constriction of the capillary vessels of the skin, impede the exit of perspirable matter, and thus destroy the equilibrium of the circulating fluids, so necessary in the economy of health." (P. 6.)

Dr. M. has had many unequivocal examples, which show that pulmonary consumption, derived from hereditary taint, can be communicated to persons who are not predisposed to the disease. He believes, however, that this occurrence never obtains unless by *direct exposure to the patient's expirations when open ulcers actually exist* in one or both lobes of the lungs, manifested by the expectoration of *pus.*

"The seeds of the disease are thus sown in the pulmonary organs; tubercles are thence generated in the parenchyma or cellular substance of the lungs by absorption: those tubercles I consider to be diseased and indurated glands attached to the lymphatic system; and, like scrofulous tumors in other parts of the body, or on the external surface, depending on enlargement of lymphatic glands, have a constant, though languid, tendency to progress to suppuration. Those tubercles also I consider as extraneous bodies; and, like other extraneous bodies differently situated, induce the suppurative inflammatory action, and communicate that excitement to the surrounding parts." (P. 10.)

The Spaniards and Italians have long been impressed with the belief that consumption is a contagious disease; and, in consequence of that opinion, they are in the habit of consuming by fire every garment which has come in contact with their departed friends. A similar opinion is also very prevalent in Scotland, and is said to be confirmed by continued observation.

For the purpose of illustrating and setting at rest that long-contested and most important question, "can consumption be communicated," the author cites a few prominent cases, selected from many others of a similar tendency, which have fallen under his own immediate observation.*

* There are but few modern pathologists of any celebrity who deny that phthisis is in a certain degree contagious. Mason Good observes, that Aristotle appeals to its contagious power as a matter of general belief among the Greeks in his day.—Rev.

Dr. Felix, of Bristol, who for several years had the medical superintendence of the depôt of prisoners at Stapleton, found that, after consumption had once made its appearance among the prisoners, there was a considerable and progressive annual increase of that class of patients; and that most of the nurses, under the age of forty, became the victims of the same disease. Laennec remarks, that in France at least pulmonary consumption does not appear to be contagious: he says, "We frequently observe among the poorer classes a numerous family sleeping in the same apartment with a consumptive patient, and a husband occupying to the last the same bed with his wife, without any communication of the disease. It is certain, however, that a disease not usually contagious may become so under certain circumstances; and we have ourselves no doubt that phthisis is occasionally communicated from one person to another by sleeping in the same bed. Dr. Myddelton remarks,

"As contagion is supposed to depend upon the morbid influence of a debilitating agent acting upon some part of the system, so I apprehend we may in this case fairly infer, by analogy, that by inhaling, from time to time, the vapour issuing from lungs in a state of actual ulceration, and discharging purulent matter, such morbid influence may be conveyed to and absorbed by lungs in the full possession of their functions, and thus propagate the disease by producing atony in the pulmonary nerves. It is upon this principle I am of opinion that we ought to account for what I consider a new fact, namely, that pulmonary consumption cannot be communicated unless open ulcers actually exist in one or both lobes of the lungs, manifested by the expectoration of pus. Upon this theory I have acted for several years, by resorting to the direct application of a tonic to the exposed organs in the incipient stage of local affection, and before any impression could have been made upon the general system. Peruvian bark and sulphate of iron are my usual resources upon those occasions where it has been too late to adopt the first precaution; and it is a consideration worth bearing in mind, that none of those persons who have embraced those preventive means have yet been visited by chronic pulmonary disease, although the usual exciting cause had often been applied." (P. 18.)

The author further adds, that in several instances the disease has been communicated to the attendants, who had disregarded his instructions.

Our readers are doubtless aware that Wilson Philip has given the name of "dyspeptic phthisis" to one species of consumption. The author of the volume before us also [redacted]

believes that we must sometimes refer the *primary* cause of pulmonary derangements to the *digestive* organs.

"In the early stage of indigestion we find little difficulty in restoring the tone of the digestive functions; but negligence, irregularity, or inattention on the part of the patient, too often protracts the disease until the second stage supervenes; the liver then sympathises with the stomach and its vitiated secretion; the bile also now undergoes a morbid change, ushering in a new train of symptoms: at this period, and not before, the lungs participate, by sympathy, in the organic affection, and thus create what may be truly termed dyspeptic phthisis. The obvious indication in such cases is to restore the lost tone of the digestive organs and hepatic gland: thus, by improving the condition of their secretions, the pulmonary symptoms will subside sympathetically, without resorting to other means, unless the disease be of long duration: in that case the lungs will become ulcerated, and require direct application, in conjunction with the plan of treatment which has been suggested. But, in dyspeptic phthisis, I have met with cases in which ulcerated lungs have yielded to direct application, when I have not been able to subdue, by general treatment, the primary disease of the digestive organs, and the consequent hepatic derangement. I have, however, more frequently observed that the lungs cannot be restored to their healthful functions, unless we can previously overcome the primary disease of the digestive organs. As such combinations of disease require very different medical treatment from that which is applicable to pulmonary consumption in its individual form, so it is of vital importance to obtain correct information in the first stage, or at the first consultation, before the patient's strength be exhausted by a combined and undefined disease." (P. 24.)

In the medical management of consumption, it is of course essential to ascertain the actual state of the pulmonary organs. Dr. M. appears to place more confidence in the following tests of the particular state of the lungs than, in our opinion, they deserve.

"We must subject the sputa, in the first instance, to the test of experiment, by dissolving it in sulphuric acid, then add about an equal quantity of water, and leave it in a quiescent state for a few hours: in the event of the lungs or the bronchial tubes being ulcerated, a precipitation of pus will take place; but, should the solution exhibit no sign of precipitation, we may confidently conclude there is no ulceration. Nor is this the only test to which we can resort. Pus may likewise be distinguished from mucus by the aid of a microscope, as the former is globular, and the latter flaky. Muriate of mercury also will afford us that information; for it will coagulate mucus, but it will not coagulate pus. The chemical test is, however, greatly to be preferred; inasmuch as the quantity of the pus precipitated within a given period can be more accurately ascertained, which will enable us to determine, with some degree of certainty, the extent of ulceration, and by which, combined with a due regard to the state of the animal functions and hepatic system, we can form a more correct prognosis.

"The experiment on the sputa should be repeated from time to time, and the precipitate weighed with care, or the quantity ascertained by a graduated measure. Its gradual diminution will be a fair criterion that the granulating process is progressing; and, when the deposition has disappeared, we may with good reason infer that the cicatrization is completed." (P. 27.)

The stethoscope is not even mentioned!

The author has "uniformly observed that the return of animal vigor is simultaneous with the decrease of purulent expectoration." We are quite sure we shall be supported by every attentive observer of this formidable disease, in objecting to so broad a statement of this opinion. A sudden decrease of the purulent expectoration not unfrequently occurs in the last stage of the disease, when the "animal vigor" of the patient is nearly exhausted. It is very true that, in some cases of presumed pulmonary consumption, the patient gains strength in proportion to the diminution of the expectoration.

Where tubercles are suspected to exist, and there are symptoms of moderate excitement, Dr. M. prefers putting the system under the actual influence of digitalis, as a more safe mean of restraining the excess of arterial action than by the abstraction of blood; unless, indeed, in plethoric habits; and even in those cases he would recommend great caution, otherwise the patient may be prematurely exhausted.

"In digitalis we have an agent possessing peculiar and adverse powers most applicable to our purpose; for, while it restrains the force of arterial action, and lessens the increased impetus of the blood through the pulmonary vessels, it gives additional energy to the absorbent system generally. I am quite aware that digitalis is going out of fashion in pulmonary diseases, and I am as fully aware of the cause. It has been given too indiscriminately, which has produced disappointment, and its enthusiastic advocates have expected it to perform impossibilities: a too sanguine friend is a dangerous enemy. But I do maintain, from my own vigilant observation, that digitalis is a safe and valuable auxiliary when judiciously administered;* and, among other advantages which we derive from its exhibition, is one of importance: by controlling the

* "When the system is placed under the actual influence of digitalis (through the medium of the stomach), it will induce vertigo and occasional nausea; but those inconveniences are avoided by subjecting that useful agent to the absorbent action of the pulmonary vessels."

pulse, we are enabled to give, with impunity, additional nourishment, which would be otherwise impracticable. Yet I do not mean to deny that cases might occur where a high degree of inflammatory action, arising from the accession of new symptoms by taking severe cold, which might sanction depletion; but such urgent symptoms are of very rare occurrence in habits so extenuated: so rare as not yet to have fallen under my observation, in the course of my attendance upon several hundred patients, who had been visited by acute symptoms, in combination with the chronic affection. Topical bleeding, by leeches or cupping, is greatly to be preferred to general bleeding, inasmuch as it does not exhaust the patient's strength to that degree; and then we bring our remedy so much the nearer to the seat of morbid action, a consideration which ought never to be lost sight of; or apply a counter-irritant by blister; or, what would be still more effective, from the greater depth of impression, frictions with tartarized antimonial ointment." (P. 31.)

The author objects to free bleeding in hemorrhage from the lungs or bronchial vessels; and no doubt the practice is too indiscriminately pursued. The cause of the hemorrhage is either the rupture of a vessel in the lungs, or (according to Laennec) exudation from the vessels of the bronchial tubes.

"If from the former, the quantity of blood discharged furnishes us with pretty correct data as to its magnitude. To meet that contingency with the promptness it often requires, I apply a styptic instanter to the mouth of the ruptured vessel, or exuding surface, as the case may be, by inhalation, and that with as much ease as well as certainty, and nearly with equal facility, as I could to a divided vessel upon the external surface; and that styptic would be, in small discharges, calcined alum and gum acaciæ; and, in more extensive hemorrhages, I employ acetate of lead in combination with cinchona, which has never disappointed me even in a solitary instance, and that too after the preceding and other remedies had proved unavailing. In my subsequent treatment I use topical tonics, or direct my attention to the healing of the coats of the ruptured vessel by the first intention, and by direct application." (P. 34.)

In order to illustrate the superior advantages to be derived from *direct* application to the lungs, the author reports and contrasts two cases which he has seen. In both, the patients

"had ruptured a vessel, the hemorrhage had ceased for several months, and both expectorated pus. The only difference in the two cases was, that my patient's hemorrhage commenced at Charleston, South Carolina, about three years before I was consulted, and had returned several times during that interval: the discharge of pus was considerable, and his strength so prostrated as to be scarcely able to quit his bed for an hour in the course of the day. The other case was under the superintendence of a professional friend, and commenced about fourteen months previously to my seeing him: he also was much emaciated. By mutual agreement, we visited each other's patients occasionally, which their situation enabled us to do without difficulty. My friend's patient was under general treatment: my patient was inhaling cinchona, myrrh, zinc, and frankincense, by which he was restored to perfect health, and the inhaler returned at the expiration of the seventh week. My friend's patient died within a month after my first visit. It is due to my friend to add, that he became a convert to direct application to the lungs by inhalation. A few weeks after my patient had ceased to require medical aid, his friend withdrew his support; and, in consequence, he became an inmate of the almshouse at Philadelphia, where I once called to see him. He informed me that he had availed himself of an opportunity to communicate his case to the physicians of the institution, (being a well-educated and intelligent man,) and on the following day they examined him with the stethoscope, and pronounced the pulmonary organs free from disease. Some months afterward he had an attack of dysentery, which had been for several weeks the prevailing disease of the house, from which he had apparently recovered, but a relapse proved fatal to him. By a post-mortem examination, (I have been informed, but I was not present at the dissection,) it was ascertained that the left lobe had been very extensively ulcerated, was much diminished in size, and the cicatrix fully and firmly formed. This case affords the most unequivocal illustration of the superior advantages of that treatment which brings the remedy into immediate contact with the diseased organ, as no medicine of any kind was conveyed into the stomach from the commencement to the termination of my attendance." (P. 36.)

A few brief observations are now offered upon the fifth and last distinction of "consumption arising from casual causes."

As, from time immemorial, it has been the uniform practice of the intelligent physician, in all diseases and in every country, to bring his remedies as nearly as he can to the seat of morbid action, Dr. Myddelton had long considered it very extraordinary and unexplained why the lungs alone, with such facilities of communication, should be exempted from the operation of so judicious a principle. The factitious gases of Beddoes and Thornton he considers inadequate to the accomplishment of their views.

The case of Mr. B. is related, who laboured under pulmonary consumption. The expectoration had been profuse. A composition of cinchona, myrrh, and zinc was conveyed *direct to the lungs* by means of an inhaler. Some improvement in the state of the patient was soon evident, and he returned the inhaler on the twenty-third day from the first visit of the author, being restored to perfect health. The only medicine conveyed into the stomach was two doses of castor oil. Two other similar cases are related.

Some observations follow upon the absorbent power of the lungs, for which we must refer to the work itself.

Dr. M. does not mean to declare that all cases of pulmonary disease, in the *advanced stage*, are to be cured by the means he recommends; but he declares that in the incipient or tuberculous stage of phthisis, or before a state of actual exhaustion has taken place in the ulcerative stage, he has lost only two patients since he has adopted the *direct* application of remedies to the diseased organs. He remarks that, in all chronic diseases, more especially in those of the lungs, from their constitutional character and insidious advance, patience is indispensable on the part of the patient, and perseverance on that of the physician, to assure a favorable result. In corroboration of the sanative effect of direct communication with diseased lungs, the following case is related:

"An intelligent farmer of Lancaster county, Pennsylvania, recommended a man in his neighbourhood, labouring under pulmonary consumption in its advanced stage, to follow the horse in a tanner's bark mill. He did so, and was cured, doubtless by inhalating the fine particles of bark floating in the atmosphere of the house which enclosed the mill. The same farmer, he also assured me, protects himself from the prevailing intermittent and remittent fevers of that section of country, by inhaling the powder of yellow bark. To what, I would ask, can we attribute those happy results? The obvious reply would be, to pulmonary absorption by direct application to those organs." (P. 68.)

The author assures us that several of the most eminent physicians in the United States have, from the mass of evidence which he has adduced, become converts to his system. We believe there is much truth in the following remarks:

"I have long been impressed with the belief that the uniformity of treatment which too often obtains in chronic disease of the lungs is, at least, injudicious. Depletion and low diet are, very properly, recommended in the incipient or inflammatory stage; but the great error in the usual practice is its continuance during the advanced or chronic stage, under the impression, I presume, of counteracting the suppurative process of other tubercles, without once attending to those coexistent ulcers which discharge pus, and sometimes calcareous concretions. This practice, surely, cannot be justified upon any rational principle. In other chronic diseases, where there is little or no excitement, we endeavour to support our patient's strength, to enable him to contend with his adversary. Ought we not, then, to adopt similar precautions in a disease which commits such regular and progressive ravages upon the animal functions of its feeble victims? From long-continued observation, I can state with confidence that consumptive patients oftener die of exhaustion than of the organic disease, and that premature exhaustion is doubtless the effect of depletion and low diet: for you all must have observed the early appearance of emaciation and loss of muscular vigor; the first from the removal of fat by the valvular lymphatic absorbents, and the latter by absorption from the muscles." (P. 77.)

The accuracy of one part of this statement is, we think, very questionable. We cannot conceive that "consumptive patients *oftener* die of exhaustion than of the organic disease," although we believe it to be occasionally the case.

Dr. M. observes, that when an *old* hepatic affection is combined with chronic pulmonary disease, in its *advanced stage*, our prognosis should be unfavorable; and he has too often seen that even the most cautious doses of mercury, administered with the view of relieving the chronic disease of the liver, has aggravated the pulmonary symptoms. In such instances he has consequently for some time discontinued the use of mercury in any form, and in those old combined cases he has since given nitric acid, and extract of dandelion and chamomile, with evident advantage.

"The lungs, in the constitutional disease, as I have already stated, may be tuberculated only, or combined with ulceration, either extensively or in a limited degree; and it is of the utmost importance to designate those stages with accuracy to assure the patient's recovery. Should the lungs be tuberculated only, the inhaler should be charged with calcined sponge, the leaves of conii and vervain, sarsaparilla, the bark of the root of mezereon, and gum ammoniac, reduced to impalpable powder, which I have uniformly observed to discuss tubercles which had not progressed to the suppurative stage. I have in some cases found that this composition excited a degree of irritation by increase of cough. Upon those rare occasions I have omitted the sarsaparilla and mezereon, and then added the powdered leaves of stramonium. In cases where the cough has assumed a spasmodic character, I have combined powdered opium with decided advantage. A diversity of remedial agents are, from time to time, submitted to the absorbent action of the vessels of the stomach and intestines; and the same principle is not only applicable, but necessary, with regard to those of the lungs, from the operation of similar causes, and regulated by practical observation.

"When ulceration of the lungs has actually taken place by the rupture of some suppurated tubercles, or from hemorrhage, I combine with some of the preceding agents (as circumstances may require) myrrh, frankincense, digitalis, cinchona, metallic oxyds, &c. It is scarcely necessary to observe, that the insoluble and fibrous particles of the remedial agents are discharged from the pulmonary organs by expectoration, and are visible in the sputa. In tuberculous cases, the inhaler should be used three times a day, at equidistant periods, and four inspirations are the proper dose, if properly conducted: but, in cases of ulceration, the most favorable time for inhaling is immediately after a copious purulent expectoration, without any regard to regular periods, in order that the subtile powder may come into direct contact with the bare excavations." (P. 82.)

To repeated depletion in the advanced stages of pulmonary consumption, Dr. M., in common with most other physicians, is decidedly opposed. Local bleeding or external irritants may sometimes be necessary.

The chief, and indeed only, object of this "preliminary dissertation" is to point out the advantage of inhaling various medical agents, in the form of impalpable powder, in pulmonary consumption. Dr. Myddelton has certainly stated enough, even in this brief lecture, to attract the serious attention of the profession to the subject; but he must allow us to add, that, if he does not quickly redeem his promise of publishing a full and perfect statement of his doctrines and his practice, he may not escape the suspicion of improper concealment. This "*avant courier* to the more elaborate offering on the altar of Humanity" has been published nearly four years.

