# Erratum in the Last Number

**Published:** 1829-06

**Authors:** 


ERRATUM *in the last Number.* The last line in p.442 to be at the top of p.439.

